# Delayed Postoperative Intracerebral Hemorrhage Associated With Oral Multikinase Inhibitor Therapy for Cancer: A Case Report

**DOI:** 10.7759/cureus.55242

**Published:** 2024-02-29

**Authors:** Vishal C Patel, Asha Krishnakumar, Edward H Yang, Andrew S Poklepovic, William C Broaddus

**Affiliations:** 1 Neurosurgery, Virginia Commonwealth University School of Medicine, Richmond, USA; 2 Medicine, University of Chicago Pritzker School of Medicine, Chicago, USA; 3 Hematology/Oncology, Virginia Commonwealth University Massey Cancer Center, Richmond, USA

**Keywords:** vascular endothelial growth factor receptor (vegf), postoperative care, anti-vegf, hemorrhage, colorectal cancer, multikinase inhibitors

## Abstract

Regorafenib is a multikinase inhibitor with anti-vascular endothelial growth factor receptor (VEGF) activity used as an antiangiogenic agent for metastatic colorectal cancer treatment and has been studied as a potential therapeutic agent for several other cancer treatments. Adverse reactions commonly reported with the use of regorafenib and similar oral multikinase inhibitors include hemorrhage, gastrointestinal fistulas, hypertension, and incomplete wound healing.

We report a case of a 59-year-old man with metastatic colorectal adenocarcinoma post-colostomy on regorafenib treatment presenting to the emergency department with altered mental status. MRI showed a left frontoparietal mass, which was resected with a left frontal craniotomy. Postoperative MRI showed a resection cavity without significant hemorrhage. He had been prescribed regorafenib preceding his hospitalization, which was continued after admission before surgery and on postoperative day 1.

Thirty-two hours after surgery, the patient exhibited sudden right-sided facial droop and right arm weakness. Imaging revealed an acute intraparenchymal hemorrhage within and adjacent to the tumor resection bed, which was managed conservatively. The patient was subsequently discharged to an inpatient rehabilitation facility.

The unusual timing of the hemorrhage suggests that the hemorrhage was due to adverse effects of regorafenib. Patients undergoing neurosurgery should have regorafenib discontinued in preparation for surgery. Similar management should be considered for other anti-VEGF medications to avoid serious complications.

## Introduction

Angiogenesis is a cancer growth hallmark and one of the underlying pathways of several malignancies. Angiogenesis often contributes to tumor survival and growth, as well as metastasis; however, associated vasculature may develop abnormally [1]. Several anti-angiogenic classes of drugs have been developed, such as monoclonal antibody inhibitors, receptor tyrosine kinase (RTK) inhibitors, soluble receptor chimeric protein, endothelial cell proliferation inhibitors, matrix metalloproteinase inhibitors, and vascular targeting drugs [2]. Pathways and growth factors underlying angiogenesis include vascular endothelial growth factor receptor (VEGFR), fibroblast growth factor receptor, platelet-derived growth factor receptor (PDGFR), and tyrosine kinase with immunoglobulin and epidermal growth factor homology domain 2 (TIE-2) [3]. Out of these growth factors and pathways, the vascular endothelial growth factor (VEGF) signaling pathway was considered to be the most important regulator of tumor angiogenesis, as it mediates survival, migration, and invasion of tumor cells [4]. One such antiangiogenic agent, regorafenib, sold under the market name Stivarga, was developed in 2012 and has been approved for the treatment of colorectal cancer, gastrointestinal stromal tumor, and hepatocellular carcinoma [5]. Regorafenib is a multikinase inhibitor and can be used for patients whose disease progresses refractory to standard chemotherapy [6]. Regorafenib inhibits RTKs of VEGFRs and is active against several angiogenic RTKs including VEGFR-1, VEGFR-2, VEGFR-3, and TIE-2 [6]. When dosed once a day for five days at 10 mg/kg to 30 mg/kg, a significant reduction of tumor microvessel area was observed in a human colorectal xenograft [7].

A review on metastatic colorectal cancer noted that in a data aggregate from multiple clinical studies including 4,800 patients, commonly reported adverse reactions with incidence greater than or equal to 30% included pain, fatigue, diarrhea, hypertension, and infection [8]. The most common serious adverse reactions of regorafenib were severe liver injury, hemorrhage, gastrointestinal perforation, and infection [8]. However, they noted that out of these studies, when looking only at controlled placebo-based studies, severe (grade 3 or greater) hemorrhage was rare as an adverse effect, with a 3% or less incidence [8]. When looking at patient groups who had their primary tumor resected versus not resected and then treated with 160 mg/day of regorafenib, the overall survival was significantly greater in patients who had surgery, at 8.2 months, versus those who did not have surgery, at 5.8 months [9]. Awareness should be raised regarding potential hemorrhagic complications in patients undergoing cranial surgery while receiving multikinase inhibitors. In this report, we present the case of a patient who was currently undergoing regorafenib treatment and whose hospital and surgical course were complicated by a delayed intracerebral hemorrhage, a unique case of sudden intracerebral hemorrhage following resection of metastatic colorectal cancer to the brain that has not been documented in the literature.

The necessary patient informed consent was obtained in this study.

## Case presentation

**Figure 1:**
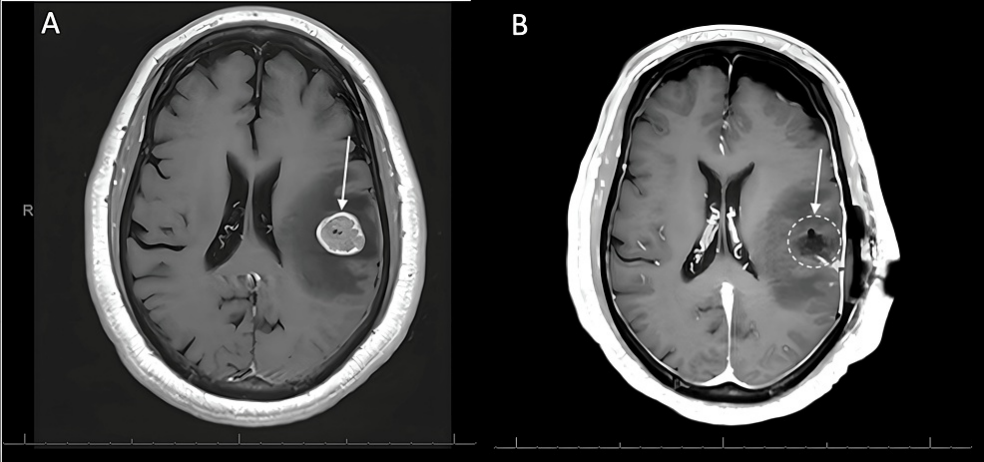
(A) Preoperative axial CT scan showing a frontoparietal mass consistent with metastasis. (B) Postoperative axial CT scan showing resection cavity.

A 59-year-old male with colorectal adenocarcinoma underwent low-anterior resection as initial treatment in October 2017 (three years before case presentation). He was currently undergoing regorafenib treatment when he presented to the emergency department (ED) with altered mental status and seizure-like activity reported by EMS. His past medical history included hypertension and congestive heart failure. In the ED, the patient was noted to have speech difficulties, right arm weakness and right facial droop. His right arm motor function was graded 4/5 on the strength scale, and subsequently improved to 5/5 after being treated with dexamethasone. The patient underwent CT imaging, which demonstrated a well-circumscribed left frontoparietal mass slightly hyperdense relative to surrounding grey matter. There was surrounding hypodensity throughout the adjacent white matter extending into the frontal and parietal lobes, consistent with a neoplastic lesion with surrounding vasogenic edema. The patient was amnestic with the event with the resolution of his speech difficulties and facial twitching, which were felt to have represented a partial seizure. The patient was admitted to neurosurgery; he was started on levetiracetam, and an MRI of the brain with and without contrast was obtained. The brain MRI demonstrated a 2.2 x 2.0 x 2.2 cm intra-axial mass located deep to the cortical surface of the post-central gyrus with significant surrounding vasogenic edema and prominent vascularity. Cerebral edema with slight effacement of the left lateral ventricle and effacement of nearby cortical sulci was also demonstrated (Figure 1A). The patient was subsequently started on high-dose dexamethasone. The patient was being treated with regorafenib at a dosage of 80 mg per day and had been followed by an outpatient oncologist. The patient started the first inpatient dose on hospital day 4. The patient consented to a left frontal craniotomy for resection of the left parietal mass with surgery planned for the following week. The operation was performed on hospital day 6 via a left frontoparietal craniotomy with intraoperative stereotactic navigation. A tubular retractor was navigated through a cortical sulcus at a safe entry point and passed to the target depth until the tumor was encountered along the anterior margin of the device corridor. Circumferential disconnection followed by piecemeal removal was used to perform a complete resection. He was transferred to the neuroscience intensive care unit post-operatively, where he was monitored for hourly neurological examinations. Postoperative MRI showed good resection with expected post-surgical changes noted in the area medial to the resection cavity (Figure 1B). Regorafenib was held due to the risk of impaired wound healing per inpatient oncology’s recommendation. The last dose was given on hospital day 7, postoperative day 1. The histology of the mass revealed adenocarcinoma, and immunostains were consistent with colorectal primary.

**Figure 2:**
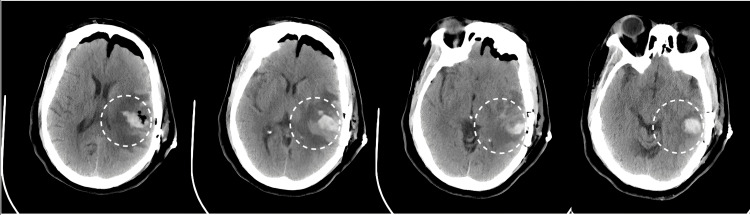
Progressive axial CT scans after the patient demonstrated a postoperative change on the neurological examination.

Twelve hours after the last dose of regorafenib, 32 hours after completing surgery, the patient demonstrated an acute change his neurologic examination. He was no longer able to follow commands on the right side and he was noted to have complete expressive aphasia. Head CT showed acute intraparenchymal hemorrhage within the tumor resection bed measuring 1.7 x 3.4 cm and extending beyond the resection cavity (Figure 2). Results of the repeat coagulation studies were within normal limits; subcutaneous deep venous thrombosis prophylaxis with heparin was held temporarily.

The patient preoperatively was on oral hydralazine and metoprolol, which were both used to control his chronic hypertension. His blood pressure monitored via radial arterial line was within the normal limits following brain surgery in our ICU; systolic pressures ranged from 109 to 164 mmHg and mean arterial pressure ranged from 72 to 106 mmHg. Blood pressure was controlled using clevidipine drip between surgery and postoperative day 1, and the patient was successfully transitioned to his home regimen of metoprolol and hydralazine. After clevidipine was discontinued, only one dose of IV labetalol was given for acute blood pressure control before the clinical examination change, leading to the discovery of the hemorrhage on the CT scan. The patient remained stable throughout the rest of his hospital course. He was started on lisinopril in addition to his current home regimen of hydralazine and metoprolol. Physical therapy and occupational therapy consulted the patient and recommended discharge to an inpatient rehabilitation facility. The patient was discharged to an inpatient rehabilitation facility on hospital day 12. The patient did not follow up with the clinic.

## Discussion

The cornerstone treatment of solitary brain metastasis is often surgical intervention [10]. Surgery is focused on an attempt at complete removal of the metastatic tumor, followed by careful attention to hemostasis to avoid postoperative hemorrhage, a well-known potential complication [10]. The hemorrhage experienced by the patient in our clinical presentation happened after the surgical resection of a metastatic intra-axial mass located deep to the cortical surface of the postcentral gyrus with significant surrounding vasogenic edema and prominent vascularity. Before resection, the patient was continued on his oral regorafenib, and the medication was only discontinued on postoperative day 1. His postoperative MRI, completed the night after surgery, demonstrated only expected post-surgical changes without evidence of excessive hemorrhage. However, 32 hours after surgical resection, the patient presented with an acute hemorrhage adjacent to the resection cavity, which extended beyond the resection cavity. The time between the last dose of regorafenib and the presentation of the acute clinical decline was 12 hours, which is a reason to suspect the regorafenib being a cause of the sudden ICH, as seen in the acute changes on the neurological examination. Of note, the mean elimination half-lives of regorafenib and one of its metabolites, M2, are similar at around 28 and 25 hours, respectively [11]. However, the mean elimination half-life of its other metabolite, M5, is around 51 hours [11]. This would mean that the medication was still in the patient's system when he presented with acute changes on the neurological examination. Medications inducing CYP3A4, such as carbamazepine, phenobarbital, rifampin, phenytoin, isoniazid, and St. John’s wort, can reduce the efficacy of regorafenib by causing a lower serum area under the curve (AUC) concentration when co-administered with regorafenib [12].

Adverse events of multikinase inhibitors, such as regorafenib, can include hypertension, thrombocytopenia, arterial and venous embolic events, and hemorrhage [13]. In particular, the risk of hemorrhage suggests that any anti-VEGF therapies should be allowed to clear from the patient’s system before undertaking surgical resection of metastatic lesions [11,12].

Aside from regorafenib, other oral multi-kinase inhibitors with anti-VEGF activity have been used in the treatment of various cancers ranging from colorectal cancer, renal cell carcinoma, breast cancer, leukemia, lymphoma, and gastrointestinal tumors. Table 1 shows oral multi-kinase inhibitors, along with known adverse reactions. Awareness of the range of side effects that each therapeutic agent presents with, along with evaluating when to administer treatment, and making sure that the treatment does not interfere with the post-operative recovery of patients undergoing surgery are crucial in reducing patient morbidity and mortality. Along with considering the use of oral multi-kinase inhibitors, more minimally invasive surgical methods, such as endoscopic removal of intracerebral hemorrhages [14] and metastatic lesions [15], can also be considered, when possible.

**Table 1:** List of currently utilized multikinase inhibitors in the treatment of cancers ALL, acute lymphocytic leukemia; BRAF, serine/threonine-protein kinase B-Raf; C-Kit, C-Kit receptor tyrosine kinase; CML, chronic myelogenous leukemia; CSFR-1, colony-stimulating factor 1 receptor; EGFR, epidermal growth factor receptor; FGFR, fibroblast growth factor receptor; Flt-3, Flt-3 receptor tyrosine kinase; HER2, human epidermal growth factor receptor 2; KIT, KIT oncogene; MET, MET oncogene; NSCL, non-small cell lung; PDGFR, platelet-derived growth factor receptor; PGF, placental growth factor; PRES, posterior reversible encephalopathy syndrome; RAF, RAF oncogene; RET, RET oncogene; TIE2, TIE2 receptor tyrosine kinase; VEGF, vascular endothelial growth factor; VEGFR, vascular endothelial growth factor receptor

Medication	Average serum half-life	Target	Complications	Approved cancers for treatment
Axitinib (Inlyta) [16]	2.5-6.1 hours	VEGFR-1, VEGFR-2, VEGFR-3	Hemorrhage, hypertension, shortness of breath, thrombocytopenia, arterial and venous embolic events	Renal cell carcinoma, /
Bevacizumab (Avastin) IV [17-19]	18.7 days (range: 11-50 days)	VEGF-A	Post-operative bleeding or wound healing complications, infusion, and hypersensitivity reactions. Severe adverse reactions include tumor-associated hemorrhage, retinal and conjunctival hemorrhage, thromboembolic events.	Glioblastoma, colorectal cancer, NSCL cancer, renal cell carcinoma, ovarian cancer, HER2-negative breast cancer
Cabozantinib (Cometriq/Cabometyx) [20]	110 hours	VEGFR-1, VEGFR-2, VEGFR-3, RET, MET	Black box warning with risk of uncontrolled bleeding and GI perforation with enterocutaneous fistula formation. Heart attack and stroke, hypertensive crisis, PRES, proteinuria.	Medullary thyroid cancer, renal cell carcinoma
Cediranib (Recentin) [21]	12-35 hours	VEGFR-2	Hypertension	Ovarian carcinoma
Lapatinib (Tykerb/Tyverb) [22]	24 hours	HER2, EGFR	QT prolongation	Breast and gastric carcinoma
Lenvatinib (Lenvima) [23,24]	28 hours	VEGFR-1, VEGFR-2, VEGFR-3, PDGFR-ɑ, FGFR, KIT, RET	Hypertension, QT prolongation, thrombocytopenia	Thyroid, renal cell, hepatocellular carcinomas
Nintedanib (Ofev/Vargatef) [25]	10-15 hours	VEGFR, PDGFR, FGFR	Small risk of hemorrhage, especially when taken while on blood thinners	Idiopathic pulmonary fibrosis, NSCL cancer
^17^Pazopanib (Votrient) [24]	27-35 hours	VEGFR, C-Kit, PDGFR	Hemorrhage, hypertensive crisis, hepatotoxicity	Clear cell renal, ovarian, NSCL cancer
Ramucirumab (Cyramza) IV [26]	14 days	VEGFR-2	Severe ascites	Hepatocellular carcinoma, gastric cancer, gastro-esophageal junction cancer, NSCL cancer, colorectal cancer
Regorafenib (Stivarga) [27,28]	28 hours (range: 14-58 hours)	VEGFR-1, VEGFR-2, VEGFR-3, TIE2, PDGFR-β, FGFR, KIT, RET, RAF	Hypertension, infection, severe liver injury, hemorrhage, and gastrointestinal perforation.	Colorectal cancer, gastrointestinal stromal tumors, hepatocellular carcinoma
Sorafenib (Nexavar) [29,30]	25-48 hours	VEGFR-1, VEGFR-2, VEGFR-3, RAF1, PDGFR, BRAF	Hypertension, liver failure. Hemorrhage very common side effect with >10% frequency	Hepatocellular carcinoma, renal cell carcinoma
Sunitinib (Sutent) [29]	40-60 hours	VEGFR-1, VEGFR-2, VEGFR-3, PDGFR, C-Kit, Flt-3, CSFR-1, Glial cell-derived neurotrophic factor receptor	Hypertension, decline in renal function, diarrhea, fatigue, and skin rash	Renal cell carcinoma, differentiated thyroid carcinoma
Tivozanib (Fotivda) [31]	4.5-5 days	VEGF-1, VEGF-2, VEGF-3, C-Kit, and PDGR kinases	Hypertension, dysphonia	Renal cell carcinoma
Vandetanib (Caprelsa) [32]	19 days	VEGFR, RET, EGFR	Reversible posterior leukoencephalopathy syndrome, hypertension, increased QTc prolongation, rash, eczema, photosensitivity reactions, pruritus, nausea and vomiting, diarrhea, and fatigue	Medullary thyroid cancer
Ziv-Aflibercept (Zaltrap) IV [33]	11.4 days after three monthly injections of 2 mg	VEGF-A, VEGF-B, PGF	Proteinuria, hypertension, intraocular inflammation, ulceration, infection, and neutropenia	Colorectal cancer

## Conclusions

We reported the case of a patient with colorectal adenocarcinoma status-post low-anterior resection being treated with regorafenib who underwent resection for a newly found frontoparietal mass consistent with metastasis. The patient subsequently had delayed acute hemorrhage following surgical resection. The patient remained on regorafenib before his scheduled resection. Shortly after tumor resection, regorafenib was discontinued; however, the patient began exhibiting acute changes in neurological examination, with subsequent CT scan showing an acute intraparenchymal hemorrhage despite discontinuation of regorafenib for at least 12 hours. This case report highlights a paucity of literature on the safety of surgical resection of intracranial metastatic disease while on oral anti-multikinase inhibitors. Furthermore, the increase in survival for patients on these newer medications may increase the detection of CNS metastases while also increasing the risk of hemorrhage associated with surgical resection.
